# Active Edible Polysaccharide-Based Coating for Preservation of Fresh Figs (*Ficus carica* L.)

**DOI:** 10.3390/foods9121793

**Published:** 2020-12-03

**Authors:** Marina Paolucci, Michele Di Stasio, Alida Sorrentino, Francesco La Cara, Maria Grazia Volpe

**Affiliations:** 1 Department of Science and Technologies (DST), University of Sannio, 82100 Benevento, Italy; paolucci@unisannio.it; 2 Institute of Food Science (ISA), National Research Council (CNR), Via Roma 64, 83100 Avellino, Italy; michele.distasio@isa.cnr.it (M.D.S.); alida.sorrentino@isa.cnr.it (A.S.); 3 Research Institute on Terrestrial Ecosystems (IRET), National Research Council (CNR), Via Pietro Castellino 111, 80131 Napoli, Italy; francesco.lacara@cnr.it

**Keywords:** fig preservation, active edible coating, antioxidant, bioactive compounds

## Abstract

Fresh figs are very sensitive to microbial spoilage, even in cold storage conditions. Thus, fresh figs are high perishable products during postharvest with microbiological decay that induces an unpleasant taste and smell due to rot, and suitable conservation methods must therefore be applied. The fruit usually is consumed fresh locally, dried, or preserved longer term in other transformed forms. A sustainable approach to extend the shelf-life of figs can be constituted by application of an edible coating able to maintain the quality of the fruit during storage. A comparison between fresh figs in a commercial preservation system, with the figs preserved in an edible coating, and an active edible coating to preserve their quality characteristics was carried out. The coating efficacy was enhanced with the addition of pomegranate peel extract at two different concentrations. The inclusion of a component with high antioxidant activity in an edible coating proved to be an excellent method for preserving the quality of this highly perishable fruit. The application of natural products, obtained from renewable sources, represents a simple and economic strategy, but also a tool capable of preserving the quality of the fruit during the postharvest storage, which is often consumed in production areas due to shelf-life problems.

## 1. Introduction

Fig fruit (*Ficus carica* L.), with probable origins in Western Asia, is an agricultural product widespread in the Mediterranean region, [1,2]. Figs are an important source of carbohydrates, mainly sugars, fibers, vitamins, minerals, and antioxidants [3].

Over the past year, the production of figs throughout the world was about 1.14 million tonnes, with approximately 90% of this production coming from the countries of the Mediterranean basin and the Middle East; Turkey contributes 20–30% to the total production, followed by other nations overlooking the Mediterranean Sea [4]. In Italy, most of the fig production comes from the southern regions, with the Campania region having the highest production, over 25% of the national production, with about 11,000 tons of fresh product per year [5].

Generally, fresh figs on the market are consumed prevalently near production areas due to the delicacy of the fruit hindering transportation [6]. Fresh figs have a short shelf-life due to microbial decay; a cold environment can extend postharvest storage life, but more suitable systems are desirable. The fruit is usually consumed fresh over three to four days or dried for longer preservation [7].

At 4–6 °C and 75% relative humidity, fresh figs remain in good condition for a few days, but have a shelf-life of only one or two days when removed from storage [8,9]. The microflora in foods originates from both the environment and manufacturing [10]. The growth and proliferation of microorganisms causes microbiological quality decay, hazardous foods, and consequent economic losses for the agrifood sector [11]. In fact, the main postharvest losses in fig fruits are rot and surrounding disease, caused by various yeasts, fungi, and bacteria; many of these are carried onto the fruits by insects such as wasps and vinegar flies. Moreover, some are able to start fermentation processes that alter the sensorial characteristics of the figs. The more representative microorganisms of the fig microflora belong to the genera *Pseudomonas, Erwinia, Flavobacterium, Xanthomonas, Pectobacterium, Enterobacter, Leuconostoc, Lactobacillus, Bacillus, Fusarium, Alternaria, Aspergillus, Hanseniaspora, Pichia*, *Saccharomyces,* and, rarely, *Enterobacteriaceae* of human origin [12,13].

To limit microbiological attachment and extend the shelf-life of figs, some preservation techniques can be applied. Modified Atmosphere Packaging (MAP) is the most applied technique [14]; although less frequent, vacuum application (VAC) can be used for the conservation of figs [15]. Among other benefits, the use of a modified or controlled atmosphere retards senescence and consequently extends the storage life of products by reducing the rate of substrate depletion. A feasible alternative to VAC and MAP could be the application of an edible coating or film, which is a preservation method used to improve food appearance and to maintain the quality of different vegetable products. In addition, edible coatings are eco-friendly, as they are obtained from renewable sources [16]. The edible coting allows control of weight loss due to the decreasing moisture of fresh fruits caused by transpiration and respiration processes [9].

Another parameter influenced by coating treatments is fruit firmness, as reported in Saki et al. [17], in which the active coating based on chitosan is an efficient alternative for firmness maintenance and shelf-life extension of fresh fig fruits. In other studies [18] the effectiveness of the chitosan-based coating was evaluated to preserve the nutraceutical traits (total polyphenol, flavonoid, antioxidant capacity, and so on) in fresh figs.

In the present study, we compared preservation systems based on the application of an edible coating and active edible coating on fresh fruits with fruits preserved by conventional procedure in order to evaluate their qualitative characteristics during the shelf-life. Moreover, in all pertinent papers, the polysaccharides matrix was constituted by chitosan, either pure or in blends, eventually with active compounds. Chitosan is a biopolymer soluble only in acidic solution and is very expansive, so we used polysaccharides, which are very widespread and low cost, such as alginate sodium and agar. These are soluble in neutral solution, with their effectiveness as coatings enhanced with the addition of pomegranate peel extract. We applied a polysaccharide edible coating or an active coating prepared as described in Laurienzo et al. [19] to fresh figs in order to establish better preservation conditions. Therefore, we analyzed qualitative parameters, pH, mechanical properties, total polyphenols, and antioxidant capacity, as well as microbial load, during 15 days of shelf-life in order to establish if the active edible coating allowed for optimal conservation of the figs and enhanced the fruits’ antioxidative capabilities. As an active biocomponent, we used pomegranate extract. Pomegranate peel extract has a high antioxidant and antimicrobial activities, as reported in previous studies [20,21].

At the best of our knowledge, this is one of the few reports on the use of an edible coating not including chitosan.

## 2. Materials and Methods

### 2.1. Materials

#### 2.1.1. Chemicals

Alginic acid (AA) sodium salt (viscosity 20–40 cps, 1% in H_2_O), and Agar (Ag) fine powder, (viscosity range 5–50 cps) were obtained from Sigma (Sigma-Aldrich, St. Louis, MI, USA). All reagents and solvents were of analytical or High Performance Liquid Chromatography (HPLC) grade.

#### 2.1.2. Plant Material

Samples of common figs analyzed in this study, (*Ficus carica* L., family *Moraceae*), free from physical damage and without microbiological contamination, were derived from a fig population located in its origin areas of San Mango sul Calore, Avellino province (Campania, Italy). Fruits were harvested in July 2019, placed in a 4 °C refrigerated box for shipment, and directly subjected to analysis. Fruits were divided into five batches, about 500 g (5 or 6 fruits), before treatments were applied, while the control (UN-FIG), consisting of untreated samples, was stored in a polypropylene container wrapped in a microperforated film.

### 2.2. Sample Preparation

The AA and Ag were dissolved in demineralized water at 100 °C under stirring. The solution concentration was 1.5% *w/v*, with a percentage ratio of the two polymers of 70/30 (considered an optimal ratio of the two components on the basis of experiments that varied the percentages of the compounds, i.e., 90/10, 80/20, 70/30, 60/40, 50/50, and vice versa). After biopolymer solubilization, the solution was maintained at 35 °C until use. The fresh figs, after careful washing and drying with kitchen paper, were dipped in the coating solution and placed on a plastic grid until coating gelation. Thereafter, the figs were placed in cardboard containers covered in microperforated polymeric material and stored at 4 °C for 15 days. For active coating, the solution AA/Ag 70/30 was added to 0.25 and 0.5% of pomegranate peel extract (PPE), prepared as described in Pagliarulo et al. [21].

### 2.3. Fig Preservation

Figs were divided into a control group and three other groups, which were subjected to treatments. Group 1: Untreated figs used as control (UN-FIG); Group 2: Figs were coated with a polysaccharide edible coating (AA/Ag 70/30) (EC-FIG); Group 3: Figs were coated with an active polysaccharide edible coating (AA/Ag 70/30 added to PPE 0.25%) (AEC_25_-FIG). Group 4: Figs were coated with an active polysaccharide edible coating (AA/Ag 70/30 added to PPE 0.50%) (AEC_50_-FIG). Analyses were performed in triplicate on all fig samples at 0, 5, 10, and 15 days.

### 2.4. Weight Measurement

The fruit weight was determined in a semianalytical balance of 0.5 to 1000 g ± 0.01 g (Gibertini electronic balance, Mod. Europe 500, Milan, Italy). Weight loss was calculated as the difference between the initial mass and the final mass, divided by the initial mass. The accumulation of weight loss was expressed as a percentage.

### 2.5. pH Determination

pH determination was carried out by a CRISON mod. 507 pH-meter equipped with type 52-00 electrodes and a type 52-32 electrode for penetration analysis.

### 2.6. Microbiological Analyses

Ten grams of fig samples were added to 90 mL of sterile Ringer solution in sterile bags (Oxoid, Basingstoke, UK) and homogenized using a Stomacher 400 (Lab Blender, Seward Medical, London, UK). Of the resulting mixture, 1 mL was taken and tenfold serial dilutions were prepared in sterile Ringer’s solution. The optimal dilutions were plated onto Plate Count Agar (PCA) plates (Thermo Fisher Scientific Inc., Oxoid Ltd., Waltham, MA, USA) to enumerate total mesophilic bacteria, on Violet Red Bile Glucose Agar (VRBGA) plates (Thermo Fisher Scientific Inc., Oxoid Ltd., Waltham, MA, USA) to detect total coliforms, and 100 μL were spread onto Yeast Extract–Peptone–Dextrose (YPD) agar plates (10 g/L yeast extract, 20 g/L peptone, 20 g/L dextrose, 20 g/L agar), to define the yeast and mold population. PCA and YPD agar plates were incubated at 30 °C respectively, for 72 h and 5 days, while VRBGA plates were incubated at 37 °C for 48 h. After incubation, the colonies were counted to enumerate the load of microbial populations in CFU/g.

### 2.7. Mechanical Analysis

Tensile tests were performed on dumbell specimens (4 mm wide and 15 mm long) by using an Instron machine (model 5564) at room temperature (RT) and a crosshead speed of 10 mm/min (average 10 samples tested). Young’s Modulus (E) was calculated from recorded curves in accordance to the American Society for Testing and Materials (ASTM) D256 standard. The evaluation of mechanical properties was performed in compressive tests. Samples of figs (with similar dimensions) were subjected to texture-o-metric tests by monoaxial compression. The samples were placed in a cylindrical plastic container (diameter 6 cm), while a spherical dart, mounted on the mobile gauge of an Instron mod. 4301 apparatus, was moved inside to halfway down (2.5 cm) at a constant speed of 1 cm/min, recording the penetration force against displacement.

### 2.8. Polar Compound Extraction

The full fig polar extracts were prepared from 5 g of sample with 80% aqueous methanol (10 mL/g of sample) by triple extraction using an Ultra-Turrax Homogenizer. The methanol extracts were obtained by centrifugation (2 min, 5000× *g*), filtered on disposable syringe 0.45 µm filters (Millipore, Billerica, MA, USA), concentrated to dryness by rotary evaporation (30 °C in a water bath), and the resulting residue was stored in a freezer (−20 °C) for subsequent analyses.

### 2.9. Total Polyphenol Content

The total phenolic content was determined by the Folin–Ciocalteu assay [22] with minor modifications [23]. Briefly, aliquots of extract or standard (20–100 μg mL^−1^ of Gallic acid) were brought at 1 mL final volume with distilled deionized water (ddH2O). Folin–Ciocalteu’s reagent (100 μL) was added to the mixture and, after 5 min, 100 μL of 7.5% Na_2_CO_3_ and 400 μL of ddH_2_O were added. The absorbance was read at 750 nm after incubation in the dark for 90 min at room temperature (RT). Total phenolic content was expressed as mg Gallic acid equivalents (GAE) × 100 g^−1^ fresh weight (FW).

### 2.10. Total Flavonoid Content

Flavonoids were measured by the aluminum chloride colorimetric assay [24]. An aliquot of extract was mixed with 1 mL of H_2_O and 75 μL of 5% NaNO_2_. After 5 min, 150 μL of 10% AlCl_3_ was added, and after 10 min, 500 μL of 1 M NaOH was added. The final volume was adjusted to 2.5 mL with H_2_O. The standard solution of Catechin (20–100 μg mL^−1^) was processed in the same way. The absorbance was measured at 510 nm and flavonoids were expressed as mg Catechin equivalents (CE) 100 g^−1^ FW.

### 2.11. Antioxidant Activity

The antioxidant activity of the full fig extracts was determined according to Von Gadow et al. [25]. Briefly, a methanolic solution of 6 × 10^−5^ M 2,2-diphenyl-1-picrylhydrazyl (DPPH) was added to an aliquot of the extract. The decrease in absorbance at 517 nm was continuously determined for 16 min. Samples were analyzed in triplicate. The radical scavenging activity percentage (%RSA) of the DPPH was calculated according to the formula %RSA = [(AC − AS)/AC] × 100, where AC is the control absorbance and AS is the sample absorbance at 16 min. The results of the antioxidant activity were expressed as EC50 (efficient concentration), that is, the extract concentration (μg mL^−1^) necessary to decrease the initial DPPH concentration by 50%.

### 2.12. Statistical Analysis

All experiments were performed in triplicate (*n* = 3) and results were expressed as mean ± standard deviation (SD). The significance between the coated group and the control groups (untreated) was measured using Student’s test of at least five determinations.

## 3. Results and Discussion

### 3.1. Weight Loss and pH Values

In fresh fruits, respiration accelerates the natural loss of fruit tissue caused by vital biological reactions after harvest. This weight loss happens through the peel by vapor pressure, leading to metabolic reactions that cause senescence, such as softening of the fresh product.

**Figure 1:**
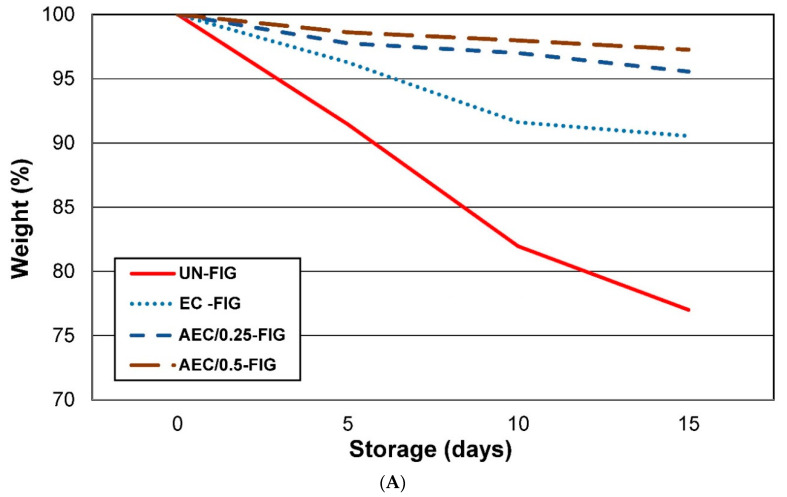

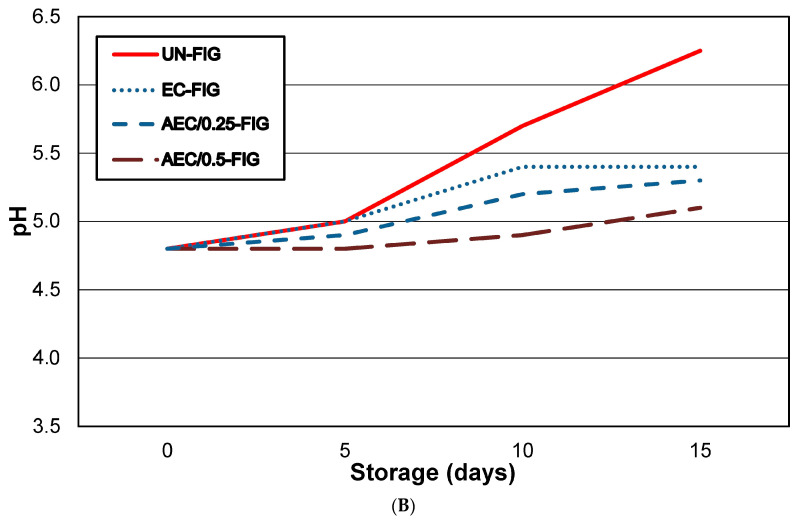
(**A**) Loss in weight (%) of fig fruits during storage at 4 °C for 15 days for all different preservation methods. (**B**) pH values of fig fruits during storage at 4 °C for 15 days for all different preservation methods.

The weight loss percentage is reported in Figure 1A. Control fruits (UN-FIG) showed the highest weight loss, while the lowest fresh mass loss was exhibited by AEC/0.25-FIG and AEC/0.5-FIG and, although to a lesser extent, by the EC-FIG samples, showing a weight loss percentage equal to approximately 9%. Weight loss of the control fruits increased gradually during the storage period due to the migration of water from the fruits to the environment, possibly attributed to the transpiration and direct evaporation through the epidermal cells [26]. In coated fruits, weight loss was reduced, suggesting that its composition promotes the formation of a network on the fruit surface which is able to contain the water loss.

The average pH values are reported in Figure 1B. The pH of figs used in this study had an average value of 4.80 at time 0. In general, UN-FIG showed an increase in pH, rising from 4.80 to 6.25 on the 15th day of storage. As reported in Mgaya-Kilima et al. [27], storage temperature and time affect pH values, while Song et al. [14] asserted that a single conservation method is not always sufficient to control this and it is often necessary to combine several methods.

EC-FIG and EC-FIG with 0.25% and 0.50% of PPE showed pH values on the 15th day of storage similar to fresh figs at day 0.

### 3.2. Microbiological Analyses

**Figure 2:**
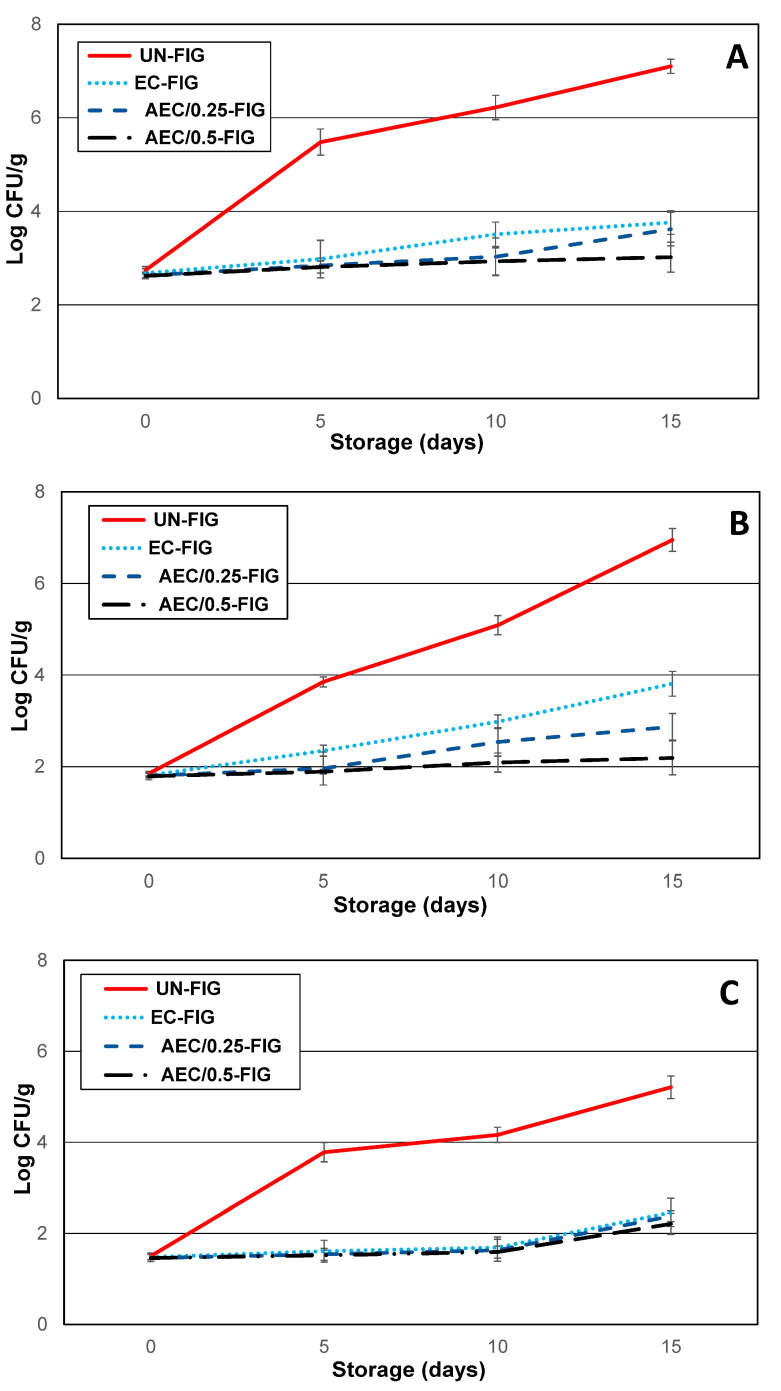
Representation of growth kinetic of spoiling bacteria, yeast, and mold populations and *Enterobacteriaceae* during storage at 4 °C for 15 days for all different preservation methods. Error bars indicate standard deviation (±SD). (**A**) TMC (total mesophilic microflora); (**B**) yeast/mold; (**C**) *Enterobacteriaceae*.

The microbiological data are reported in Figure 2A–C, showing a continuous increase in the microflora population in the UN-FIG samples. In detail, the total mesophilic microflora (TMC) developed throughout a storage time of 7.1 Log10 UFC/g, with an initial TMC value of 2.5 Log10 UFC/g in UN-FIG. The same trend was shown by yeast and mold populations and also total bacterial coliforms (reported in the Figure as the Enterobacteriaceae family). This behavior may be related to the ability of the aerobic and facultative anaerobes microorganisms to present as components of the indigenous fig microflora that can grow in high and low levels of O_2_ or high levels of CO_2_. Our experimental data were in accordance with other studies [7]. In the EC-FIG, AEC/0.25-FIG, and AEC/0.5-FIG groups, the various microbial groups showed lower population loads, with TMC values of 2.6 Log10 UFC/g at 0 day and 3.7–3.0 Log10 UFC/g at day 15. The yeast and mold values were 1.8 Log10 UFC/g at 0 day and 3.8–2.2 Log10 UFC/g at 15 days, and Enterobacteriaceae exhibited values of 1.5 Log10 UFC/g at 0 day and 2.4–2.2 Log10 UFC/g at 15 days. We assumed that the indigenous fig microflora did not find suitable conditions to grow during storage, as suggested also from other authors [7]. This different growth capability could be the direct consequence of the technological characteristics of the edible coating and the experimental conditions. In particular, in AEC/0.25-FIG, AEC/0.5-FIG samples, the percentages of active components with antimicrobial proprieties [9] influenced the microflora growth. Both AEC/0.25-FIG and AEC/0.5-FIG samples showed minimal increases in TMC, yeast, molds, and total coliform bacteria after ten days, less than one logarithmic order, perhaps due to the decrease in PPE antimicrobial efficiency. These data confirmed that coating application with antimicrobial characteristics could enhance the microbial safety of fruits [28].

### 3.3. Mechanical Properties

The firmness of many fruits such as figs is a useful parameter to evaluate the state of maintenance of the qualitative characteristics [29]. The loss of fruit firmness is associated with the action of cell wall degrading enzymes, which hydrolyze starch to soluble sugars and protopectin to water-soluble pectin [17] alongside microbial decay.

As a matter of fact, the deformability, the resistance to break, and the compactness of a food as observed during mastication and the energy related to these operations induce a pool of sensorial stimuli in the consumer which contribute to the judgment on the quality of the food. In this study, we carried out mechanical tests in order to quantify the consistency of the figs as a function of time and type of storage. Samples of figs were compressed in an Instron type dynamometer using a monoaxial load, and the results were recorded as penetration force against displacement.

**Figure 3:**
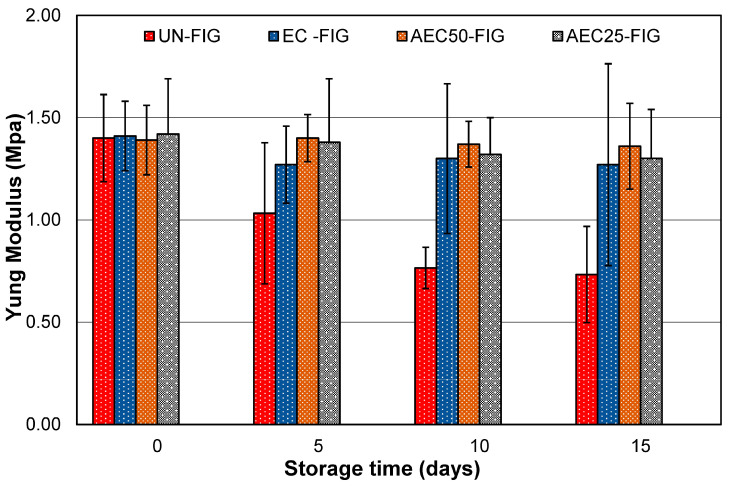
Compression tests of fig fruits during storage at 4 °C for 15 days for all different preservation methods.

After five days (Figure 3), UN-FIG showed a strong diminution of the compression strength, while EC-FIG still maintained a very high strength. The most significant results were obtained in AEC/0.25 and AEC/0.50 after 15 days of storage. In both groups, the mechanical strength was practically unchanged respective to the initial values.

As a result, the edible coating combined with the active component positively contributed to the maintenance of firmness in figs by reducing water loss and fruit senescence and decreasing cell wall degradation through the inhibition of microbial propagation.

### 3.4. Total Polyphenols, Flavonoids and DPPH

#### 3.4.1. Total Polyphenols

Fresh fruit and vegetables are highly perishable products, and water loss and postharvest decay account for most of their losses. During the postharvest life of fruit and vegetables and the different technological treatments to which they are submitted in order to extend their shelf-life, some changes in secondary metabolism occur. The decrease in the levels of phenolic compounds might be due to the breakdown of cell structure leading to senescence during storage. These metabolic changes in phenolic compounds, often coupled with the activity of polyphenol oxidase, are responsible for some phenomena affecting the quality of stored fruits and vegetables [29].

Various feasible technological measures can be adopted to reduce such losses and improve shelf-life. These include harvesting, handling, maturity, low temperature storage and environmental control (controlled/modified atmosphere, hypobaric storage), irradiation, use of chemicals and fungicides, and packaging techniques [30]. Our study confirmed that phenolic metabolism is dependent on a preservation system remaining at the same storage temperature.

As shown in Table 1, during the 15 day storage at 4 °C, the total phenolic content in UN-FIG decreased from 95.27 to 84.91 mg Gallic acid equivalents (GAE)/g fresh weight (FW). In the coated samples, the values changed, respectively, from 95.37 to 90.21 mg GAE/g FW in EC-FIG, from 95.67 to 102.94 mg GAE/g FW in AEC/0.25-FIG, and from 90.21 to 110.04 mg GAE/g FW in AEC/0.5-FIG.

The results show a reduction of approximately 20–25% for UN-FIG, while a small decrease was observed in the EC-FIG group. EC-FIG, with 0.25–0.50% of PPE, recorded a moderate increase in total polyphenols, in particular when the active component was present at 0.50%. A significant difference (*p* < 0.05) was shown after 15 days between UN-FIG and AEC/0.25-FIG, UN-FIG and AEC/0.50-FIG and AEC/0.25-FIG using the unpaired t-test. The values showed that the active edible coating was able to enhance the content of phenolic acids. The increase in polyphenols found in EC-FIG containing PPE was probably due to the gradual release of the polyphenols from the pomegranate extract trapped in the polymer matrix. A comparison with available literature data showed a decrease in polyphenol contents of 19% using other polysaccharide edible coatings [18], while our experiments over a similar range of time (first 10 days) exhibited PPE values for AEC-FIG of 0.25–0.50% increasing from 2.3% to 11.0%, respectively.

#### 3.4.2. Total Flavonoids

The total flavonoid contents in all samples slightly decreased during the first five days of cold storage, when fruit rot was not evident at this time. Thereafter, the method of conservation had a significant impact on the evolution of flavonoid contents (Table 2).

In fact, UN-FIG showed the highest loss of total flavonoid content, followed by EC-FIG, while no significative changes were recorded for AEC/0.25-FIG and AEC/0.50-FIG. UN-FIG flavonoid contents were significantly different (*p* < 0.01) from AEC/0.25-FIG and AEC/0.5-FIG after 15 days. A comparison with available literature data showed a decrease in flavonoid contents of 12.6% using other polysaccharide edible coating [18], while our experiments over a similar range of time exhibited practically unchanged PPE values for AEC-FIG of 0.25% and 0.50%.

#### 3.4.3. Antioxidant Activity

The DPPH radical scavenging assay is among the most frequently used methods for evaluating antioxidant activity, and is based on the electron donation of antioxidants to neutralize DPPH radicals. The reaction is accompanied by a colour change of the DPPH solution measured at 517 nm, and the discolouration acts as an indicator of the antioxidant efficacy. The antioxidant activity by the DPPH scavenging method is often reported as EC50, which is defined as the amount of antioxidant (expressed as µg of total polyphenols) necessary to decrease the initial DPPH concentration by 50% (EC50: efficient concentration).

In Table 3, we report the antioxidant activity as EC50, at the beginning and at the end of the storage time, measured every five days.

The antioxidant activity decreased over time, with UN-FIG samples showing a major decrease, EC-FIG exhibited a minor decrease, and an almost constant value exhibited by AEC/0.25-FIG and AEC/0.50-FIG. The samples treated with the active coating showed values higher than those of UN-FIG at the end of storage, a result attributable to the use of the pomegranate peel extract that expresses high antioxidant activity [18,30].

As for the polyphenolics compounds, but also for the antioxidant activity, we found that storage with an active coating significantly affected the antioxidant activity of figs.

Literature data reported that the antioxidant activity of figs depends on cultivars, phenolic compounds [31,32], and a combination of different molecules with synergic and antagonistic effects. In our case, we always used the same cultivar; therefore, the decrease in antioxidant activity in UN-FIG was related to the decrease in phenolic compound [32,33] contents and other antioxidant components of the figs during storage time.

## 4. Conclusions

Our results showed the ability of an active polysaccharides coating to preserve the microbial, antioxidant, and mechanical properties of fresh figs. The inclusion of a component with high antioxidant and antimicrobial activities in an edible coating proved to be an excellent method for preserving the quality of highly perishable fruits, such as figs. Therefore, we propose that the packing method used in this work could preserve some qualitative parameters that change with increasing postharvest time, such as chemical and microbiological characteristics, texture, and antioxidant properties.

Moreover, the application of natural products, obtained from renewable sources, represents a simple and economic strategy, but also a tool capable of preserving the quality of the fruit due postharvest storage, which is often consumed in production areas due to shelf-life problems. Therefore, an active coating could be used to extend the storage life of highly perishable fruits such as figs, even if more in-depth studies are required for successful commercialization in the agrifood industry.

## Figures and Tables

**Table 1:** Changes in total polyphenol contents (mg Gallic acid equivalent (GAE)/100 g fresh weight (FW)) occurring after 5, 10, and 15 days of storage at 4 °C with different packaging.

Storage Time	Sample			
	UN-FIG	EC-FIG	AEC/0.25-FIG	AEC/0.50-FIG
Day 0	95.27 ± 4.1	95.37 ± 0.31 ^(ns)^	94.8 ± 2.3 ^(ns)^	94.70 ± 0.46 ^(ns)^
Day 5	91.17 ± 0.39	93.17 ± 0.40 ^(ns)^	94 ± 4.4 ^(ns)^	97.95 ± 0.74 ^(ns)^
Day 10	89.27 ± 0.83	92.1 ± 52.31 ^(ns)^	97 ± 1.8 *	105.14 ± 0.31 *
Day 15	84.91 ± 0.81	90.21 ± 0.30 *	102 ± 5.1 *	107.04 ± 0.48 **

Changes in total polyphenols comparing polysaccharide edible coating (EC-FIG) vs. control (UN-FIG), active polysaccharide edible coating (AEC)/0.25-FIG vs. UN-FIG, and AEC/0.5-FIG vs. UN-FIG. Significant differences were determined with a *t*-test. *p*-value > 0.5 = nonsignificant (ns); * *p*-value < 0.5; ** *p*-value < 0.01.

**Table 2:** Changes in total flavonoid contents (mg Catechin equivalents (CE)/100 g FW) occurring after 5, 10, and 15 days of storage with different packaging.

Storage Time	Sample			
	UN-FIG	EC-FIG	AEC/0.25-FIG	AEC/0.50-FIG
Day 0	41.2 ± 2.0	41.5 ± 1.5 ^(ns)^	42.7 ± 6.4 ^(ns)^	43.3 ± 2.0 ^(ns)^
Day 5	42.3 ± 3.7	40.2 ± 5.3 ^(ns)^	42.8 ± 5.9 ^(ns)^	44.8 ± 3.2 ^(ns)^
Day 10	38.6 ± 4.3	38.2 ± 4.6 ^(ns)^	41.9 ± 8.3 **	44.1 ± 4.1 **
Day 15	34.7 ± 7.4	39.3 ± 5.1 *	42.0 ± 2.8 **	43.6 ± 7.5 **

Changes in total flavonoids comparing EC-FIG vs. UN-FIG, AEC/0.25-FIG vs. UN-FIG, and AEC/0.5-FIG vs. UN-FIG. Significant differences were determined with a *t*-test. *p*-value > 0.5 nonsignificant (ns); * *p*-value < 0.5; ** *p*-value < 0.01.

**Table 3:** Changes in antioxidant activity (EC50) occurring during shelf-life after 5, 10, and 15 days of storage at 4 °C with different packaging.

Storage Time	Sample			
	UN-FIG	EC-FIG	AEC/0.25-FIG	AEC/0.5-FIG
Day 0	8.2 ± 0.6	8.3 ± 0.5 ^(ns)^	8.1 ± 0.2 ^(ns)^	8.5 ± 0.4 ^(ns)^
Day 5	7.6 ± 0.2	8.0 ± 0.7 ^(ns)^	8.1 ± 08 ^(ns)^	8.3 ± 0.3 ^(ns)^
Day 10	6.9 ± 0.6	7.6 ± 0.3 *	7.9 ± 0.2 *	8.1 ± 0.1 **
Day 15	6.3 ± 0.3	7.5 ± 0.1 *	7.8 ± 0.8 *	8.0 ± 0.9 **

Changes in antioxidant activity comparing EC-FIG vs. UN-FIG, AEC/0.25-FIG vs. UN-FIG, and AEC/0.5-FIG vs. UN-FIG. Significant differences were determined with a *t*-test. *p*-value > 0.5: nonsignificant (ns); * *p*-value < 0.5; ** *p*-value < 0.01.
